# Combined Methods (Formal Adjusted Indirect Comparison, Meta-Analysis and Principal Component Analysis) Comparisons of the Safety and Efficacy of Ambrisentan, Bosentan, and Sildenafil in the Patients With Pulmonary Arterial Hypertension

**DOI:** 10.3389/fphar.2020.00400

**Published:** 2020-04-03

**Authors:** Xinmei Li, Te Li

**Affiliations:** Department of Pharmacy, Fuwai Yunnan Cardiovascular Hospital, Kunming, China

**Keywords:** ambrisentan, bosentan, sildenafil, principal component analysis, formal adjusted indirect comparison

## Abstract

**Background:**

Three oral drugs (ambrisentan, bosentan, and sildenafil) have been widely used to treat patients with pulmonary arterial hypertension (PAH). 1) There are no studies that directly compare the safety and efficacy of these three drugs. Existing studies could not meet the physician’s need to select the most beneficial drugs for patients. 2) Principal component analysis is mainly used for scale analysis and has not been reported in clinical field. 3) When the results of the indirect meta-analysis were not satisfactory, no new solutions have been proposed in existing meta-analysis studies.

**Methods:**

The overall process of this study is divided into 4 steps 1) literature search and data extraction; 2) principal component analysis; 3) common reference-based indirect comparison meta-analysis; 4) formal adjusted indirect comparison.

**Results:**

Nine randomized controlled trials (RCTs) experiments and eight long-term experiments were selected. The main influencing factors are mortality, 6-min walk distance (6MW), mean pulmonary arterial pressure (PAP), cardiac index (CI) by principal component analysis. There was no significant heterogeneity among the indirect meta-analysis of three drugs. But in the formal adjusted indirect comparison 1) the level of PAP of sildenafil group (60.5 ± 22.35, 220) was higher than that of the other three groups, placebo (53.5 ± 17.63, 507) (p < 0.001), ambrisentan (49.5 ± 15.08, 130) (p < 0.001), and bosentan (54.6 ± 118.41, 311) (p < 0.001); 2) the level of CI of sildenafil group (54 ± 18, 311) was higher than that of the other three groups, placebo (2.7 ± 1.09, 518) (p < 0.001), ambrisentan (2.5 ± 0.75, 130) (p < 0.001), and bosentan (2.5 ± 1.06, 333) (p < 0.001). In addition, sildenafil significantly improved the survival rate comparing with ambrisentan and bosentan.

**Conclusions:**

The results of this study suggest that sildenafil might be more suitable for long-term treatment of PAH patients than ambrisentan and bosentan. In order to enable clinicians to draw conclusions more quickly and directly in the data-rich literature, we suggest the use of principal component analysis combined with formal adjusted indirect comparison to compare the efficacy and safety of drugs.

## Introduction

Pulmonary arterial hypertension (PAH) is a progressive disease, which may involve multiple clinical conditions and can complicate the majority of cardiovascular and respiratory diseases. It is mainly characterized by elevated pulmonary arterial pressures (PAP) and vascular resistance. The increase of PAP, PAP ≧25 mmHg at rest, could be assessed by right heart catheterization. Research showed that the survival rates was 68.0% in 1 year, 38.9% in 3 years, and 20.8% in 5 years (Zhi-Cheng et al., 2007). In other words, PAH is a serious chronic life-threatening disease.

In the past decade, traditional supportive therapy (oral anticoagulants, diuretic, O_2_, digoxin) has failed to improve the patient survival rate, while specific drug therapy has become a more widely accepted long term treatment modality in recent years. As recommended by the 2015 ESC/ERC guidelines, drugs including ambrisentan, bosentan, and sildenafil were class IA for efficacy of oral monotherapy drug (Nazzareno et al., 2016). Among them, bosentan and ambrisentan could antagonize the endothelial dysfunction, in which endothelin-1 has been found to be overexpressed in PAH patients (Giaid et al., 1993). Bosentan is the first synthetic molecule of its class and a dual endothelin-1 receptor type A and B antagonist. Ambrisentan preferentially binds type A. The third drug, sildenafil, is a selective inhibitor of phosphodiesterase type 5. Eventually, the treatment of PAH with the above three drugs could result in vasodilation through pathway such as endothelin (ET) pathway and nitric oxide (NO) pathway (Galie et al., 2004; Benedetta et al., 2005; John et al., 2005).

Recently, although some meta-analyses and systematic reviews of individual drugs have been published, in which they have typically been compared with placebo. However, there have been no large randomized controlled trials comparing the drugs to one another reported, while too many indicators of the safety and efficacy are used. In the absence of directly comparable studies, it is difficult for general practitioners and cardiologists to directly select the most beneficial and safe treatment. In addition, indirect meta-analysis of the three drugs has not been reported in PAH patients. The purpose of this study is to combine three analysis methods, such as formal adjusted indirect comparison, meta-analysis, and principal component analysis, to analyze the treatment options for PAH patients. We sought to provide a direct and quick analytical method to assist patients and clinicians decide in clinical practice.

## Materials and Methods

**Figure 1:**
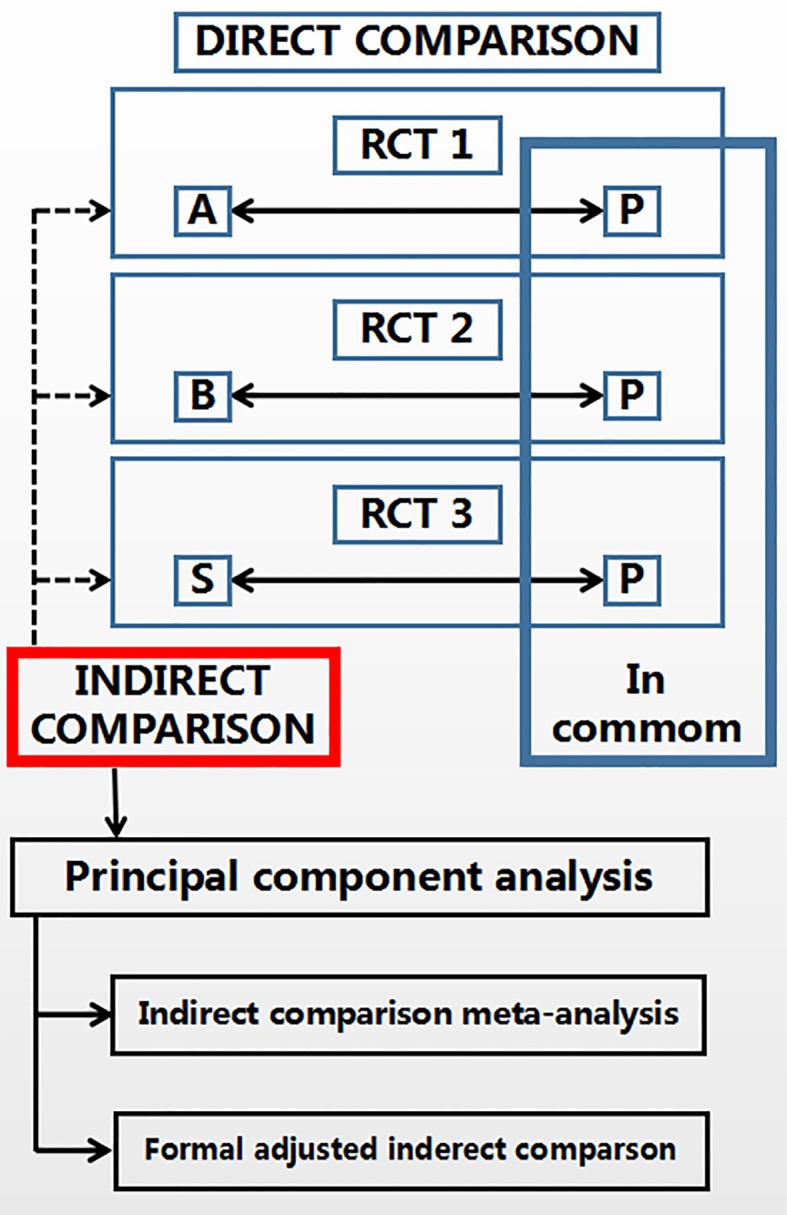
Study flow diagram.

The overall process of this study is divided into four steps: 1) literature search and data extraction; 2) principal component analysis; 3) common reference-based indirect comparison meta-analysis; 4) formal adjusted indirect comparison (**Figure 1**).

### Literature Search and Inclusion Criteria

We primarily performed an exhaustive search of studies examining the efficacy and safety of ambrisentan, bosentan, and sildenafil in patients with PAH. The literature was searched using MEDLINE, EMBASE, CNKI, WANFANG, and Cochrane Library (up to May 2019). The following keywords and subject terms were used in the searches: ambrisentan, bosentan, sildenafil, pulmonary arterial hypertension. Randomized controlled trials (RCTs) which met the following criteria were included in this study: 1) the study compared oral monotherapy drug, ambrisentan, bosentan, and sildenafil, with a placebo for PAH; 2) the study provided endpoints for the clinical efficacy and safety; 3) the publication was in Chinese or English. The exclusion criteria were as follows: 1) Studies conducted *in vitro* experiments and animal studies, 2) the study used combination multidrug therapy such as iloprost, and 3) the study included duplicated data or did not contain adequate data for inclusion. Safety outcomes were mortality. As recommended by the 2015 ESC/ERC guidelines, efficacy outcomes were as follows: 1) 6-min walk distance (6MW), 2) mean pulmonary arterial pressure (PAP), 3) cardiac index (CI), 4) Pulmonary vascular resistance index (PVR), and 5) mean right atrial pressure (RAP).

### Data Extraction and Quality Assessment

According to the recommended guidelines of the Cochrane Handbook for Systematic Reviews, the extraction form, created with Microsoft Excel, included initial author’s name, year of publication, study site, study design, mean age of participants, and sample size, dose, length of follow-up, and efficacy and safety outcomes and so on. We quantified the methodological qualities of the studies using Jadad scores. These assessments were based on the following 3 criteria: 1) whether the randomization method was appropriate, 2) whether double blindness was mentioned in the trial and the trial was appropriately performed, and 3) whether the number of patients that withdrew or dropped out, and the reasons for this, were clearly stated.

The two authors carried out independent reviews. Discrepancies between the reviewers were resolved through consensus. The reviewers assessed the methodological quality of each study by using the risk of bias method recommended by the Cochrane Collaboration.

### Statistical Analysis

We chose dichotomous primary outcomes to have hard outcome measures of treatment efficacy. Analyses were conducted using Excel, R 3.6.0 (principal component analysis), StataSE 15 (common reference-based indirect comparison meta-analysis), and GraphPad Prism 6 (formal adjusted indirect comparison).

#### Principal Component Analysis

The purpose of principal component analysis was to describe the relationship among many indicators with a small number of principal components (Wangzong and Jiahong, 2014). In this study, the software R 3.6.0 was utilized for the principal component analysis of the extracted indicators such as mortality, 6MW, PAP, CI, PVR, and RAP. When the sum of the influencing factors is ≧ 85%, the influencing factors are considered as the principal component.

#### Common Reference-Based Indirect Comparison Meta-analysis

Differences among ambrisentan, bosentan, and sildenafil were assessed by odds ratio (OR) with 95% confidence intervals (CIs). The random-effect model was used to calculate OR (Dersimonian and Laird, 1986). The possibility of publication bias was estimated by funnel plots. Heterogeneity among studies was evaluated by calculating *p*-value and the *I* ^2^ measure of inconsistency, which was considered significant if *p* < 0.10 or *I* ^2^ > 50%. All calculations were carried out using StataSE 15. Results were considered as statistically significant when the *p* value was < 0.05. Common reference-based indirect comparisons were performed using the method suggested by Xiantao Z (Xiantao and Xuequn, 2017): the indirect comparison of ambrisentan, bosentan, and sildenafil was adjusted by the results of their direct comparisons with placebo.

#### Formal Adjusted Indirect Comparison

According to the group of placebo and drug administration, the mean, sd, and n values of main indicators from principal component analysis were formal adjusted by formula 1, formula 2, and formula 3 (Jiahong and Tianhe, 2010). The combination formula 1 of two data **(Supplementary Text 1)**:

$$\text{M}=\frac{\left({\text{N}}_{1}{\text{M}}_{1}+{\text{N}}_{2}{\text{M}}_{2}\right)}{\left({\text{N}}_{1}{+\text{N}}_{2}\right)}$$

The combination formula 2 of two data:

$$\mathrm{SD}=\sqrt{\frac{\left({\text{N}}_{1}-1)\text{S}{\text{D}}_{1}^{2}+({\text{N}}_{2}-1){\text{SD}}_{2}^{2}+\frac{{\text{N}}_{1}{\text{N}}_{2}}{{\text{N}}_{1}+{\text{N}}_{2}}({\text{M}}_{1}^{2}+{\text{M}}_{2}^{2}-2{\text{M}}_{1}^{2}{\text{M}}_{2}^{2}\right)}{{\text{N}}_{1}+{\text{N}}_{2}-1}}$$

The combination formula 3 of two data:

$$\text{N}={\text{N}}_{1}+{\text{N}}_{2}$$

And multiple *t* tests and graphs of each safety indicators of drugs were applied in GraphPad Prism 6. Comparing *p*-values between groups and results were considered as statistically significant when the *p*-value was < 0.05.

## Result

**Figure 2:**
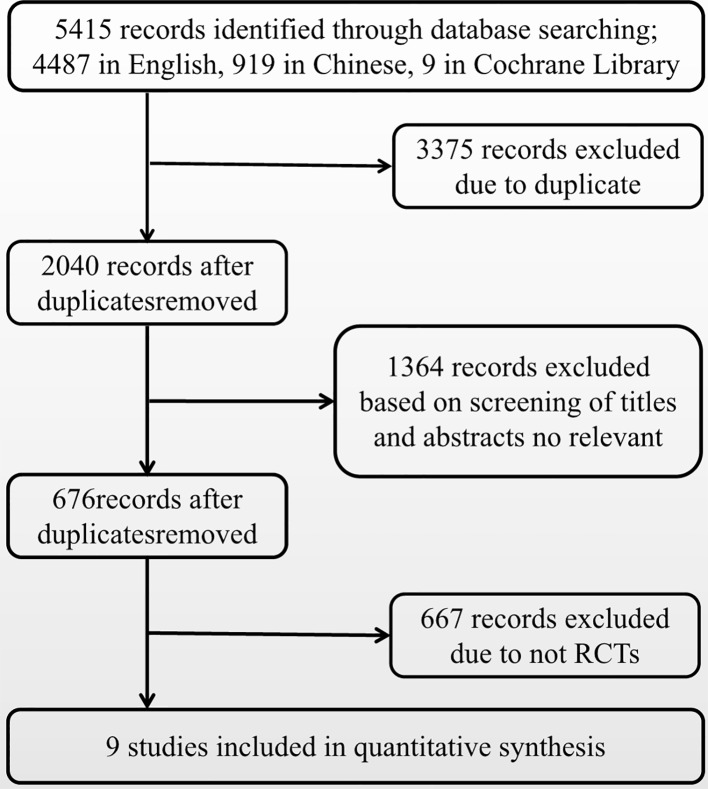
Literature screening flow diagram.

**Figure 2** presents a flowchart describing the trial screening and selection procedure. After the search strategy, nine reports were included in this systematic review (Channick et al., 2001; Lewis et al., 2002; Humbert et al., 2004; Nazzareno et al., 2006; Galiè et al., 2008; Barst et al., 2010; Robyn et al., 2011; Carmine et al., 2017; Nazzareno et al., 2018). A total of six studies compared bosentan versus placebo, two studies compared sildenafil versus placebo, and one study compared ambrisentan versus placebo. **Table 1** summarizes the methodological quality of the included trials.

**Table 1:** Characteristics and the quality assessment of the nine studies included.

Study, year	Drug	Abbr.	Patients(N)	Term	Random sequence generation	Allocation concealment	Blinding	Incomplete outcome data addressed	Selective reporting	Other bias
Nazzareno et al., 2018	Ambrisentan	A1-1	201	12 weeks	Yes	Yes	Unclear	Yes	Yes	Unclear
		A1-2	192	12 weeks	Yes	Yes	Unclear	Yes	Yes	Unclear
Channick, 2001	Bosentan	B1	32	20 weeks	Yes	Yes	Unclear	Yes	No	Unclear
Barst et al., 2010		B2	14	16 weeks	Yes	Yes	Unclear	No	Unclear	Unclear
Galiè, 2008		B3	185	6 months	Yes	Yes	Yes	Yes	Yes	Unclear
Lewis, 2002		B4-1	213	16 weeks	Yes	Yes	Unclear	Yes	Unclear	Unclear
		B4-2	33	16 weeks	Yes	Yes	Unclear	Yes	Unclear	Unclear
Humbert et al., 2004		B5	33	16 weeks	Yes	Yes	Unclear	Yes	Unclear	Unclear
Nazzareno et al., 2006		B6	54	16 weeks	Yes	Yes	Yes	Yes	Unclear	Unclear
Robyn, 2011	Sildenafil	S1	234	16 weeks	Yes	Yes	Unclear	Yes	Unclear	Unclear
Carmine et al., 2017		S2	86	24 weeks	Yes	Yes	Unclear	Yes	Unclear	Unclear

### Principal Component Analysis

According to the result (**Table 2** ) from R 3.6.0, the product of the eigenvalue corresponding to each principal component and proportion of variance is used to calculate the comprehensive model of principal component. The principal component formula **(Supplementary Text 2):**

F=F1×0.3995+F2×0.2372+F3×0.1526+F4×0.1375+F5×0.00650+F6×0.00080

Sum=(0.3995+0.2372+0.1526+0.1375)×100%=92.85%>85.00%

**Table 2:** Importance of components by principal component analysis.

	Mortality	6mw	PAP	CI	PVR	RAP
Standard deviation	1.5483	1.1931	0.9569	0.9085	0.6246	0.2197
Proportion of Variance	0.3995	0.2372	0.1526	0.1375	0.0650	0.0080
Cumulative Proportion	0.3995	0.6367	0.7893	0.9269	0.9919	1.0000

The principal components are F_1_(mortality), F_2_(6mw), F_3_(PAP), and F_4_(CI), which will be used to do the indirect comparison. However, F_5_(PVR) and F_6_(RAP) are screened and removed without further discussion.

### Common Reference-Based Indirect Comparison Meta-analysis

#### Statistical Analysis of Efficacy Outcomes (6MW, PAP, CI)

**Figure 3:**
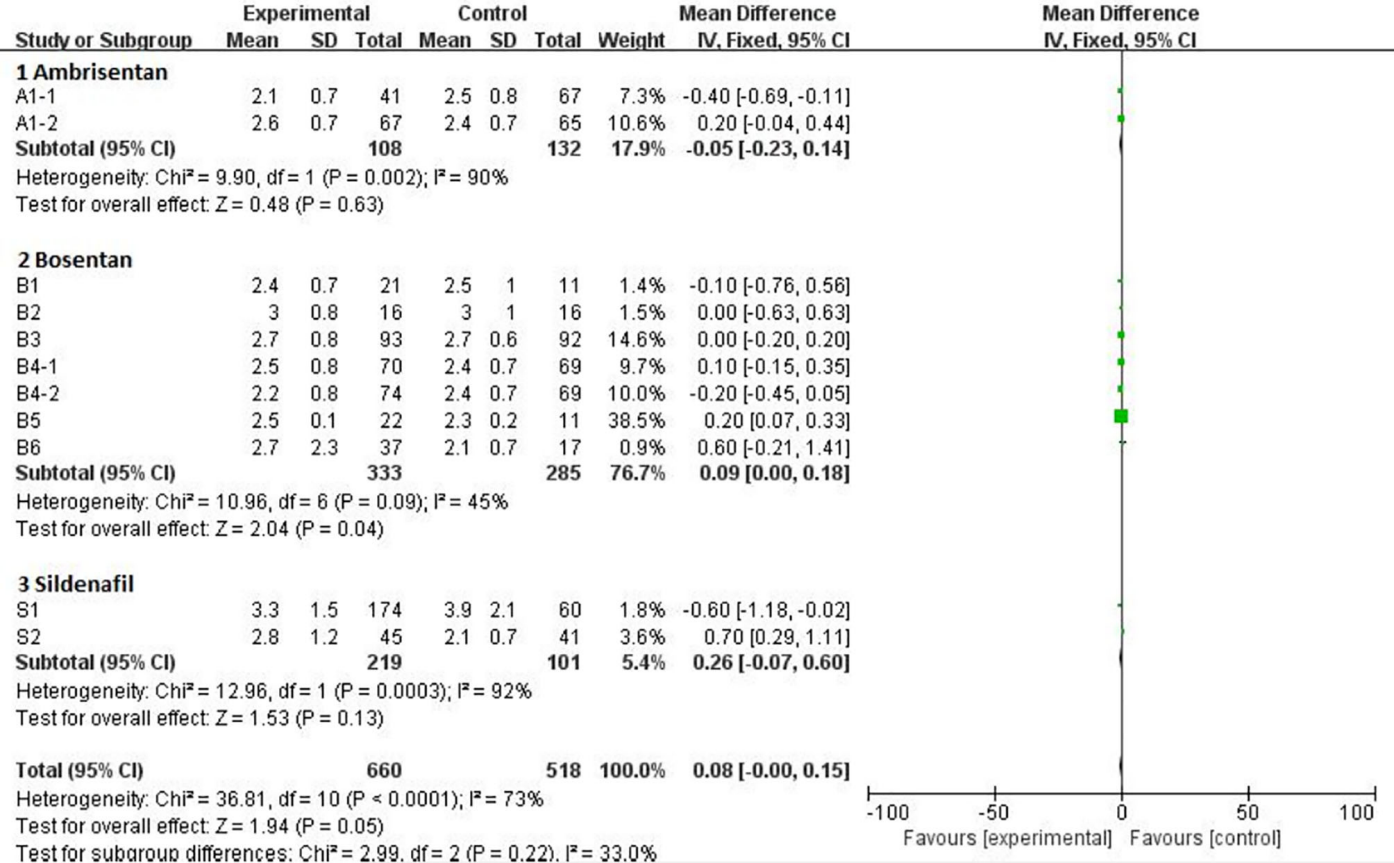
Comparison ambrisentan, bosentan, and sildenafil versus placebo, outcome of meta subgroup in CI.

Mean Difference (MD) was available for the 6MW, PAP, and CI trials. The statistics of the pooled analysis of MD using the random-effects model is showed in **Table 3**. Only the data from the CI trail, including treatment with ambrisentan, bosentan, and sildenafil, showed favorable results with an MD of −0.7 (95% CI, −1.11 to −0.29). Further subgroup analysis of CI revealed that the heterogeneity mainly came from all three drugs (**Figure 3**). The MD of ambrisentan, bosentan, and sildenafil subgroup is −0.05 (95% CI, −0.32 to 0.14), 0.09 (95% CI, 0 to 0.18), and −0.26 (95% CI, −0.07 to 0.15), respectively. The overall difference in the CI group was mainly from the ambrisentan and bosentan subgroups.

**Table 3:** Mean difference (MD) result of meta-analysis for the 6MW, PAP, and CI trials by software RevMan.

	MD	CIs (95%)		Chi2	df	P	I2 (%)	Z	P
6MW	−5.38	−15.66	4.89	5.48	7	0.6	0	1.03	0.3
PAP	−0.87	−2.7	0.95	16.61	9	0.06	46	0.94	0.35
CI	−0.7	−1.11	−0.29	25.39	10	0.005	61	2.37	0.02

The indirect comparison OR of the 6MW, PAP, and CI for ambrisentan versus bosentan, ambrisentan versus sildenafil, bosentan versus sildenafil is in **Table 4**. But there was no significant heterogeneity between the indirect comparison of ambrisentan, bosentan, and sildenafil.

**Table 4:** The indirect meta-analysis of the 6MW, PAP, and CI for ambrisentan versus bosentan, ambrisentan versus sildenafil, bosentan versus sildenafil by software STATA.

		Exponential Statistic OR	CIs (95%)		Chi2	P
6MW	A vs B	0.112	0	2933.422	0.178	0.673
	A vs S	0	0	5.437	2.6	0.107
	B vs S	0.003	0	60.005	1.294	0.255
PAP	A vs B	2.466	0	2.99E+08	0.009	0.924
	A vs S	0.513	0	20323.13	0.015	0.902
	B vs S	1.077	0	40807.864	0	0.989
CI	A vs B	1.208	0	1.60E+36	0	0.996
	A vs S	0.985	0.272	3.566	0.001	0.982
	B vs S	0.988	0	0.274	1.27	0.985

#### Statistical Analysis of Safety Outcomes on Long Term (Mortality)

**Figure 4:**
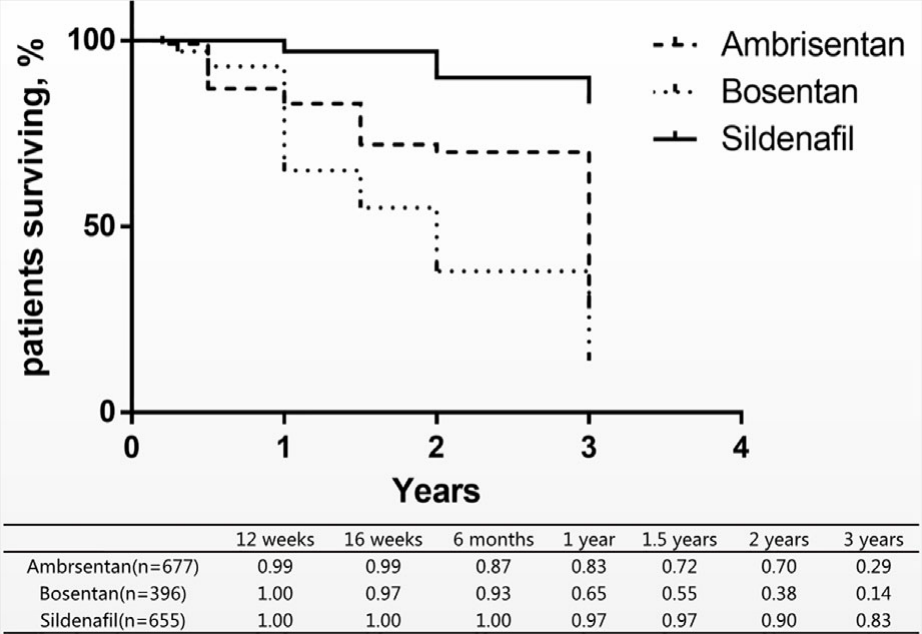
Patients surviving and survival rate of three drugs treatment within 3 years.

Since no deaths occurred in the sildenafil groups, ambrisentan and bosentan could not be directly compared with sildenafil in the short-term mortality. Therefore, we combined eight long-term studies of mortality of three drugs for comparison of the differences (**Figure 4**) (Lewis et al., 2000; Antonio et al., 2006; Ronald et al., 2009; Shannon et al., 2010; Wouter et al., 2010; Michele et al., 2012; Shunji et al., 2012; Robyn et al., 2014). In these studies, patients with PAH were treated with ambrisentan, bosentan, and sildenafil from 12 weeks to 3 years. A research assessed the survival rates at 68.0% in 1 year and 38.9% in 3 years (Zhi-Cheng et al., 2007). Comparison with this research, ambrisentan and sildenafil increased the 1-year survival rate by 83% and 97%. Only sildenafil increased the 3-year survival rate by 83%.

#### Formal Adjusted Indirect Comparison

**Figure 5:**
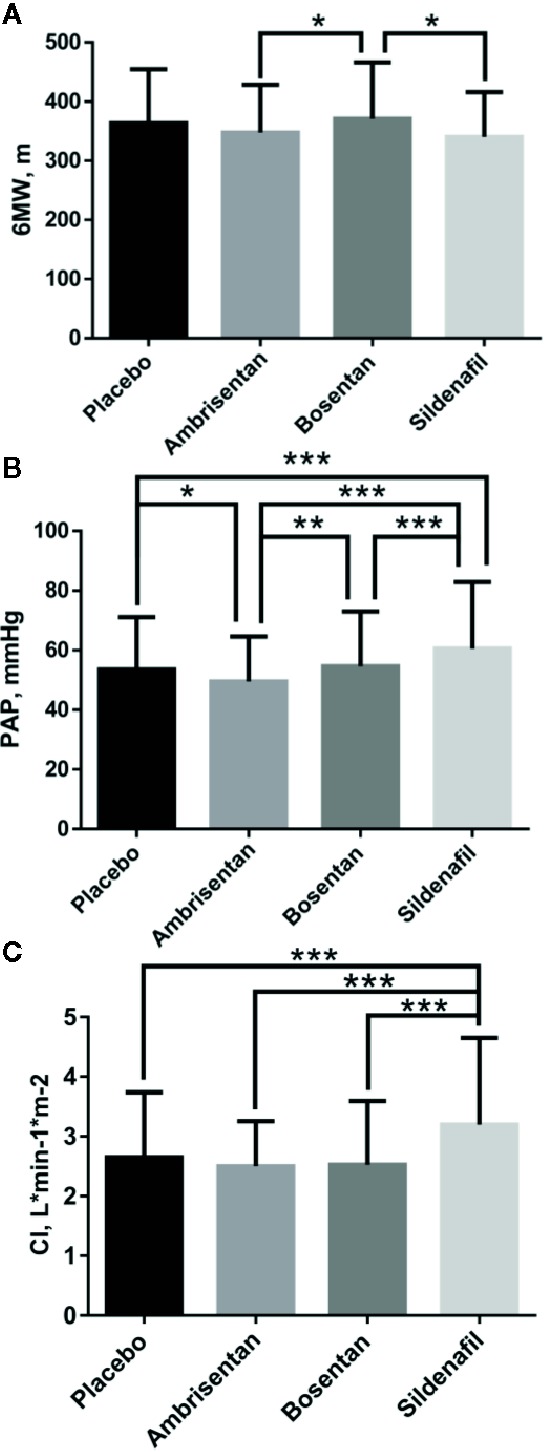
Comparative differences of multiple t test of 6MW **(A)**, PAP **(B)**, and CI **(C)**. Statistical analysis was performed using multiple t test. *P < 0.05; **P < 0.01; ***P < 0.001.

After combined Mean, SD, and N by formula 1, formula 2, and formula 3 (**Table 5**), multiple t test showed more differences in 6MW, PAP, CI indicators than the indirect comparison meta-analysis (**Figure 5**). The difference between the four groups was very small in the comparison of 6WM indicator. The level of bosentan group (371 ± 95, 307) was slightly higher than that of ambrisentan group (347 ± 80, 130) (p < 0.05) and sildenafil group (340 ± 76, 45) (p < 0.05). In the comparison of PAP data, the level of sildenafil group (60.5 ± 22.35, 220) was higher than that of the other three groups, placebo (53.5 ± 17.63, 507) (p < 0.001), ambrisentan (49.5 ± 15.08, 130) (p < 0.001), and bosentan (54.6 ± 118.41, 311) (p < 0.001). In the comparison of CI data, the level of sildenafil group (54 ± 18, 311) was higher than that of the other three groups, placebo (2.7 ± 1.09, 518) (p < 0.001), ambrisentan (2.5 ± 0.75, 130) (p < 0.001), and bosentan(2.5 ± 1.06, 333) (p < 0.001).

**Table 5:** Formal adjusted results (Mean, SD, N) of the 6MW, PAP, and CI by formula 1, formula 2, and formula 3.

	Placebo			Ambrisentan			Bosentan			Sildenafil		
	Mean	SD	N	Mean	SD	N	Mean	SD	N	Mean	SD	N
6MW	363.5174	91.2368	451	347.7846	80.9527	130	371.0963	95.2210	307	340.0000	76.0000	45
PAP	53.5020	17.6330	507	49.5462	15.0812	130	54.6645	18.4091	311	60.5118	22.3599	220
CI	2.6502	1.0873	518	2.5031	0.7539	130	2.5291	1.0636	333	3.1973	1.4551	219

## Discussion

### Discussion of the Safety and Efficacy of Three Drugs

Currently, the pathways for the oral treatments of PAH are mainly divided into two types: ET pathway and NO pathway. ET and NO are two of the most important vasoconstrictor and vasoconstrictor factors. Under normal conditions, both factors work together to maintain the normal state and function of blood vessels (Kawanabe and Nauli, 2011). However, in pulmonary hypertensive disorders, it is reported that ET-1 receptor type A is abnormally activated, NO synthase gene expression, and NO signaling are reduced (Stephen et al., 2007). Bosentan blocks ET-1 receptor type A and B, and ambrisentan blocks ET-1 receptor type A. Sildenafil mainly enhances NO and cyclic guanosine phosphate signaling pathways. In this study, sildenafil significantly improved the survival rate comparing with ambrisentan and bosentan as shown in **Figure 4**, and the value of PAP and CI was higher than ambrisentan and bosentan as shown in **Figure 5**. This finding suggests that the higher value of PAP and CI may imply lower mortality. However, some researchers suggest that PAP only provides little prognostic information and CI is a robust indicator of hemodynamics (Sitbon et al., 2005; Nickel et al., 2012). Some even suggest that the estimated PAP is not relevant for therapeutic making (Raymond et al., 2010). Therefore, the finding of this study is very interesting and awaits independent confirmation. In addition, the finding of this study may imply that choosing NO pathway may be more effective and safer than the ET pathway in the PAH treatment. There are 10 drugs in the ET pathway and 14 drugs in the NO pathway from drugbank.ca. Therefore, this finding also awaits further confirmation.

As shown in **Figure 5**, the comparison of 6MW values shows no significant difference. Some researchers assert that may due to the placebo effect in the RCTs (Carmine et al., 2017). 6WM is a submaximal exercise test and influenced by several factors, including sex, age, need for O_2_, and motivation. The recent researches also showed no relationship between magnitude of exercise improvements and survival (Nazzareno et al., 2009; Alejandro et al., 2010). The results of the 6MW analysis are consistent with the results of the existing published studies.

### Discussion of the Combined Methods

Principal component analysis was mainly used in scale analysis. This study, to the best of our knowledge, is the first to use principal component analysis to analyze clinical trial data. This study confirms that it is very feasible to screen out the main components from multiple factors and can be used in clinical field. This method could be extended by clinical data researchers to effectively screen out important disease-related biochemical information, especially those who analyze multiple laboratory biochemical results. This study has proved the practicality of this method in clinical research through experiments, which is of great significance. As shown in **Table 6**, when there are too many efficacy indicators, clinical researchers might randomly select these indicators in clinical trials, which would bring great difficulties to data analysis. We suggest that clinical researchers could use principal component analysis to screen published effective indicators when designing studies, which might be conducive to forming norms and even guidelines for indicators in this research area. If this method could be widely used in the clinical field, on the one hand, it might shorten the time for doctors to analyze the results of clinical trials, on the other hand, it might reduce the cost of patients for unimportant examination items.

**Table 6:** Determination of six indicators in 11 studies.

		Mortality		6MW, m			PAP, mmHg			CI, L•min-1•m-2			PVR, wood units			RAP, mmHg		
		n	N	mean	sd	n	mean	sd	n	mean	sd	n	mean	sd	n	mean	sd	n
Placebo	A1-1	2	67	342	73	73	50.0	15.0	67	2.5	0.8	67	10.85	6.48	67	8.0	5.0	67
	A1-2	4	65	343	86	65	51.0	13.0	65	2.4	0.7	65	12.14	7.24	65	7.0	5.0	65
	B1	0	11	355	82	11	56.0	10.0	11	2.5	1	11	11.78	5.38	11	9.9	4.1	11
		B2	0	8	353	170	14	38.0	7.0	16	3	1	16	5.44	2.36	16	NA	NA
	B3	1	92	431	91	92	52.3	16.0	92	2.7	0.6	92	10.06	4.61	92	7.5	5.1	92
	B4-1	2	69	344	76	69	53.0	17.0	69	2.4	0.7	69	11.00	6.75	69	8.9	5.1	69
	B4-2	0	11	344	76	69	53.0	17.0	69	2.4	0.7	69	11.00	6.75	69	8.9	5.1	69
	B5	0	11	NA	NA	NA	NA	NA	NA	2.3	0.2	11	13.13	1.93	11	NA	NA	NA
	B6	0	17	366	68	17	72.1	19.4	17	2.1	0.7	17	17.94	7.56	17	5.0	3.7	17
	S1	0	60	NA	NA	NA	59.0	22.0	60	3.9	2.1	60	15.00	10.00	60	8.0	5.0	60
	S2	0	41	348	67	41	57.2	21.9	41	2.1	0.7	41	15.70	9.90	41	10.5	5.1	41
agents	A1-1	1	134	341	78	67	51.0	16.0	67	2.6	0.7	67	11.40	5.81	67	9.0	6.0	67
	A1-2	2	127	355	84	63	48.0	14.0	63	2.4	0.8	63	11.64	8.40	63	8.0	5.0	63
	B1	0	21	360	86	21	54.0	13.0	21	2.4	0.7	21	11.20	5.31	21	9.7	5.6	21
	B2	1	6	370	122	12	31.0	6.0	16	3	0.8	16	4.90	2.25	16	NA	NA	NA
	B3	1	93	438	86	93	52.5	18.9	93	2.7	0.8	93	10.49	6.64	93	6.9	4.5	93
	B4-1	3	144	326	73	70	53.0	14.0	70	2.5	0.8	70	11.05	5.15	70	9.7	5.4	70
	B4-2	2	22	333	75	74	57.0	17.0	74	2.2	0.8	74	14.59	10.94	74	9.9	6.5	74
	B5	2	22	NA	NA	NA	NA	NA	NA	2.5	0.1	22	11.84	1.30	22	NA	NA	NA
	B6	0	37	332	83	37	77.8	15.2	37	2.7	2.3	37	21.41	8.82	37	6.1	3.4	37
	S1	0	174	NA	NA	NA	63.0	22.0	174	3.3	1.5	174	20.00	15.00	174	8.0	5.0	174
	S2	0	45	340	76	45	51.1	21.4	46	2.8	1.2	45	11.70	9.10	45	8.4	4.7	45

NA means there is no relevant value in the references.

This study first proposes the formal adjusted indirect comparison could be used as alternative method, when the results of the indirect meta-analysis were not satisfactory. The main advantage of meta-analysis software lies in the visualization of forest maps. However, when the difference between the placebo group and the treatment group is small, the visualization effect is significantly weakened, as shown in **Figure 3** of this study. It could be clearly known through this study that formal adjusted indirect comparison resulted in more intuitive data results than indirect meta-analysis. Formal adjusted indirect comparison are graphically visualized using the software GraphPad Prism 6, which is easier to manipulate than meta-analysis software. This might be very friendly to researchers who may not have a background in meta-analysis, and can help them speed up the time to analyze data, especially for doctors who treat acute illnesses.

## Conclusion

We indirectly compared the effectiveness and safety of ambrisentan, bosentan, and sildenafil, for the first time, and found that sildenafil might be more suitable for long-term treatment of PAH patients than ambrisentan and bosentan, because it can significantly improve the survival rate. In order to enable clinicians to draw conclusions more quickly and directly in the data-rich literature, we suggest the use of principal component analysis combine with formal adjusted indirect comparison to compare the efficacy and safety of drugs.

## Data Availability Statement

The raw data supporting the conclusions of this manuscript will be made available by the authors, without undue reservation, to any qualified researcher.

## Author Contributions

XL: Data analysis, discussion, the meta-analysis methods and results, data extraction and quality control was performed. TL: The data extraction and quality control.

## Conflict of Interest

The authors declare that the research was conducted in the absence of any commercial or financial relationships that could be construed as a potential conflict of interest.
