# Supplementary material for: Combined Methods (Formal Adjusted Indirect Comparison, Meta-Analysis and Principal Component Analysis) Comparisons of the Safety and Efficacy of Ambrisentan, Bosentan, and Sildenafil in the Patients With Pulmonary Arterial Hypertension
Source: Front Pharmacol. 2020 Apr 3;11:400. doi: 10.3389/fphar.2020.00400 (PMC7145892; doi:10.3389/fphar.2020.00400)
Supplement: Supplementary file 1 [file DataSheet_1.docx]

Supplementary text 1. The acronyms and explanation for formula 1, formula 2 and formula 3.

M: the calculated mean value

N_1_: numbers of group 1

N_2_: numbers of group 2

M_1_: mean of group 1

M_2_: mean of group 2

SD: the calculated standard deviation value

SD_1_: standard deviation of group 1

SD_2_: standard deviation of group 2

Supplementary text 2. The acronyms and explanation for the principal component formula:

F: total factor

F_1_: mortality

F_2_: 6mw

F_3_: PAP

F_4_: CI

F_5_: PVR

F_6_: RAP)
